# Cost-effectiveness of watchful waiting versus immediate emergency department transfer after epinephrine autoinjector use in Canada

**DOI:** 10.1186/s13223-025-00951-w

**Published:** 2025-01-22

**Authors:** Yiwei Yin, Moshe Ben Shoshan, Marcus Shaker, Matthew Greenhawt, Kate M. Johnson

**Affiliations:** 1 Collaboration for Outcomes Research and Evaluation, Faculty of Pharmaceutical Sciences, University of British Columbia, 2405 Wesbrook Mall, Vancouver, BC V6T 1Z3 Canada; 2 McGill University Health Centre, Montreal, QC Canada; 3 Section of Allergy and Immunology, Dartmouth-Hitchcock Medical Center, Lebanon, NH USA; 4 Section of Allergy and Clinical Immunology, Children’s Hospital Colorado, University of Colorado School of Medicine, Aurora, CO USA; 5 Division of Respiratory Medicine, Faculty of Medicine, University of British Columbia, Vancouver, BC Canada

**Keywords:** Anaphylaxis, Watchful waiting, Cost-effectiveness analysis

## Abstract

**Background:**

Until recently, immediate emergency department (ED) transfer after food-related anaphylactic reactions was recommended regardless of symptom resolution following use of an epinephrine autoinjector (EAI). We evaluated the cost-effectiveness of delayed ED transfer after EAI use in non-medical settings (watchful waiting) compared to immediate ED transfer among pediatric patients with food allergies in Canada.

**Methods:**

We developed a probabilistic Markov model of individuals starting at age of one year who are at risk of severe food-related allergic reactions requiring epinephrine. We evaluated medical costs (in 2022 Canadian dollars) and quality-adjusted life years (QALY) of each strategy over a 20-year horizon. In the base case, we assumed a tenfold increase in food allergy fatality for patients under watchful waiting, which we increased to 100- to 1,000-fold in sensitivity analysis. The analysis was conducted from the Canadian healthcare system perspective with a 1.5% annual discount rate and a willingness-to-pay (WTP) threshold of $50,000 per QALY.

**Results:**

Immediate ED transfer following EAI use resulted in a decreased risk of food allergy fatality of 9.2 × 10^− 5^ over 20 years, which is equivalent to < 1 fatality per 200,000 patient-years. Watchful waiting resulted in cost savings of $1,157 per patient and a QALY loss of 7.28 × 10^− 4^; an incremental cost per QALY saved of $1,589,854. The incremental cost per death prevented with immediate ED transfer was $12,586,613. Watchful waiting remained cost-effective in all sensitivity and scenario analyses, except under extreme increases in fatality risk of 500-fold and 1,000-fold.

**Conclusions:**

Watchful waiting for symptom re-occurrence following EAI administration in non-medical settings is cost-effective.

**Supplementary Information:**

The online version contains supplementary material available at 10.1186/s13223-025-00951-w.

## Background

Anaphylaxis is the most severe manifestation of an allergic reaction, affecting more than 2% of the North American population annually with increasing emergency department (ED) visits [1]. Food allergy is the primary cause of anaphylaxis in children. An estimated 8% of Canadians, or approximately 3 million people, have food allergy, and 42% of patients with food allergy report a previous allergic reaction, including anaphylaxis [2, 3]. Fortunately, fatal food allergy anaphylaxis reactions are rare, occurring in fewer than one in every 500,000 patients with a food allergy; however, they are unpredictable and death can occur within 30 min after exposure without prompt treatment [4–6]. The mainstay of treatment for anaphylaxis has been epinephrine, traditionally administered intramuscularly via an epinephrine autoinjector (EAI), though a nasal form is now available. Delayed administration of epinephrine is a risk factor for refractory and prolonged reactions, intensive care unit admission, and death [7, 8].

There is widespread underutilization of EAI among children in pre-hospital settings. Almost half of pediatric anaphylaxis cases occur at home, one-third of which occur under adult supervision [9]. However, use of an EAI is delayed in the majority of cases, and is associated with severe outcomes and an increased risk of fatal anaphylaxis when epinephrine is administered more than one hour following allergic reactions [10, 11]. Current anaphylaxis management guidelines recommend pre-hospital epinephrine treatment, which leads to prompt resolution of symptoms in most cases. Studies suggest that underutilization of EAI worsened in 2020–2021 due to the COVID-19 pandemic, when fewer patients sought emergency medical care [12]. Before the most recent updates to anaphylaxis management guidelines in 2023 [13], all anaphylactic cases were required to be transferred to the ED for 4–6 h of observation and management regardless of pre-hospital EAI use [14, 15]. Recent studies suggest a universal requirement for emergency medical services may negatively impact EAI use due to patients’ reluctance to visit the ED [12]. This impact is particularly pronounced in rural and remote communities, where there are logistical and financial burdens to accessing an ED [16].

During the COVID-19 pandemic, there was a shift towards early use of an EAI at home and home-based observation, rather than immediate activation of emergency services and transportation to a healthcare facility [13, 17]. This change was reflected in the 2023 updates to anaphylaxis management guidelines conducted by the U.S.-based Joint Task Force on Practice Parameters [13]. Their recommendation for delayed activation of emergency services among patients with resolved or resolving symptoms following EAI administration was supported by an evidence-based cost-effectiveness analysis conducted in the U.S. healthcare setting, which reported a cost of more than $1.3 billion (US dollars) per peanut allergy fatality avoided through immediate emergency medical services activation, mainly owing to the low rates of symptom re-occurrence and fatality following epinephrine administration [18]. Similar changes to Canadian position statements on the use of EAI in pre-hospital settings have been proposed, but evidence of cost-effectiveness in the Canadian healthcare system is lacking [19].

In light of recent revisions to policies for anaphylaxis management, we assessed the economic outcomes and risk of fatality of non-mandatory ED transfer among Canadian pediatric patients whose symptoms promptly resolve without re-occurrence following EAI administration. Specifically, we evaluated the cost-effectiveness of ‘watchful waiting’ following a severe allergic reaction requiring epinephrine, in which an EAI is administered at home followed by observation, and the patient is only transferred to the ED if symptoms do not resolve, versus a policy of immediate ED transfer regardless of EAI use.

## Methods

This study was reported in accordance with the consolidated health economic evaluation reporting standards (checklist in Supplemental Table 1) [20]. The target population was Canadian patients with food allergies older than one year of age. We assumed all patients in our study could potentially develop a severe allergic reaction, which is defined as non-self-resolving IgE-mediated anaphylactic reaction anaphylaxis requiring EAI intervention, or a reaction subjectively or objectively judged by the caregiver to be severe and necessitate EAI use. We analyzed the incremental costs and quality-adjusted life years (QALYs) of two post-anaphylaxis strategies over a 20-year time horizon: 1) immediate ambulance transfer to the ED regardless of EAI use (‘immediate ED transfer’) for all patients with severe allergic reactions, and 2) a wait-and-monitor approach following at-home EAI administration for those with resolved/resolving reactions (‘watchful waiting’). In the watchful waiting strategy, all patients with non-resolving symptoms, either because of a recurrent allergic reaction or because they did not have access to an EAI, were transported to the ED immediately without any waiting period. We evaluated the incremental cost-effectiveness ratio (ICER) and incremental net monetary benefit (INMB) using a willingness-to-pay (WTP) threshold of $50,000 per QALY gained [21]. The base case analysis was conducted from the healthcare system perspective. All costs were reported in 2022 Canadian Dollars (CAD), with a discount rate of 1.5% per annum applied to both costs and effects [22]. This study used simulated data and was exempt from institutional review board approval and informed consent. The model was implemented in R version 4.3.1 (https://www.r-project.org) with heemod package version 0.16.1 [23]. Model code is publicly available at https://github.com/resplab/Allergy_EAI_CEA.

### Model structure

**Fig. 1:**
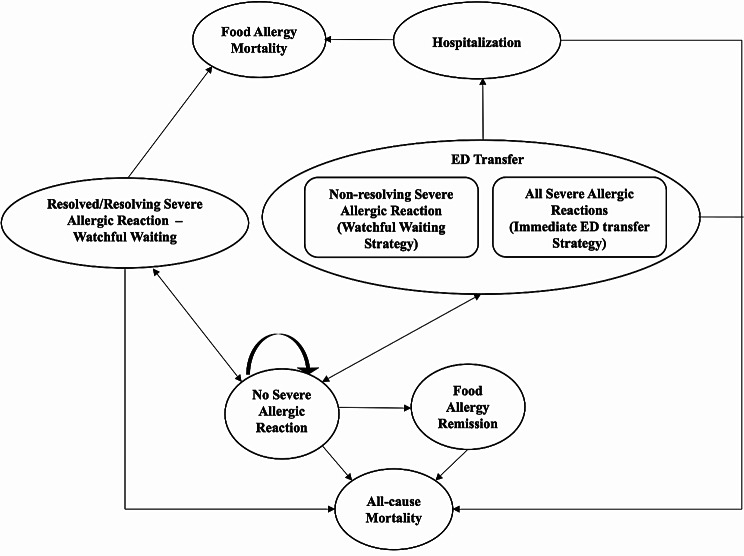
Model structure. ED: emergency department

Patients with food allergy transitioned between the following seven health states in our model: 1) no severe allergic reaction, 2) at-home EAI use followed by watchful waiting of resolved/resolving severe allergic reaction, 3) ED transfer, 4) hospitalization, 5) food allergy mortality, 6) food allergy remission, and 7) all-cause mortality (Fig. 1). A severe allergic reaction was defined as an anaphylactic reaction following accidental allergen exposure that required epinephrine. Our model used daily cycles to reflect the transient nature of a severe allergic reaction.

Patients entered the simulation in the ‘no severe allergic reaction’ state at one year of age. They remained in this state until they (a) achieved food allergy remission, (b) suffered a severe allergic reaction due to accidental exposure, or (c) died from causes unrelated to food allergies. Management following severe allergic reaction differed between comparator strategies. In the watchful waiting strategy, patients administered an EAI at home and returned to the no severe allergic reaction state after one day if symptoms resolved or were resolving within 15 min of EAI administration and did not recur within 72 h (biphasic reaction). Patients who did not carry an EAI or in whom a biphasic reaction occurred were immediately transferred to the ED. In the immediate ED transfer strategy, all patients with severe allergic reaction were transferred to the ED regardless of EAI use and symptom resolution. Following an ED visit, patients could be admitted to the hospital for an additional day or return to the no severe allergic reaction state. The probability of ED-to-hospital admission was the same for both strategies, as our model assumed all hospitalizations occurred through ED assessment rather than direct hospital admission. There was a risk of food allergy mortality outside of hospital in the severe allergic reaction watchful waiting state and in-hospital from the hospitalization state. All-cause mortality could occur from any state.

### Probability and events

Model parameters are given in Table 1. The annual probability of severe allergic reaction was 0.087, which was based on the number of severe allergic reactions observed from 2016 to 2021 in the Cross-Canada Anaphylaxis Registry (CCARE) [24], assuming a 7% prevalence of food allergy among Canadians ≤ 18 years [25]. The probability of an ED visit following severe allergic reaction was 100% in the immediate ED transfer strategy, and 14% in the watchful waiting strategy, which was calculated by adding the proportion of Canadians with known food allergy ≤ 18 years who reported not carrying an EAI (9.4%) [26], to the probability of biphasic reaction after anaphylaxis in a meta-analysis (4.6%) [27]. Among the 8.7% of patients who were transferred to the ED with severe allergic reaction, the probability of hospitalization was 1.68 × 10^− 3^. As this parameter could not be found in the literature, it was determined through calibration so that the probability of hospitalization multiplied by the probability of food allergy fatality among hospitalized patients (4.5 × 10^− 3^, determined from US hospital admissions data [28]) equaled the 6.9 × 10^− 7^ annual probability of food allergy fatality among all patients with food allergy in the US [5, 28]. We assumed the same probability of hospitalization and food allergy fatality among patients transferred to the ED in the watchful waiting strategy. The annual risk of food allergy fatality outside of hospital after severe allergic reaction through watchful and waiting strategy was 0.031 annually, which was determined by subtracting the in-hospital risk of food allergy fatality (4.5 × 10^− 3^), weighted by the 0.14 probability of ED visit and subsequent probability of hospitalization (1.68 × 10^− 3^), from the overall food allergy fatality rate (6.9 × 10^− 6^). Details of the calculations are shown in Supplemental Table 2.

**Table 1:** Model parameters

Parameter	Base case	DSA (lower/upper)	PSA distribution	Source
Baseline age	1 year			
Cycle length	1 day			
Discount rate, % per annum	1.5			CADTH [22]
Annual probability of severe allergic reaction following accidental exposure	0.087	0.070/0.10	Beta (α = 66, β = 696)	Analysis of CCARE registry data [24, 25]
Probability of patient with known food allergy having an EAI^a^	0.906		Beta (α = 8.5, β = 0.88)	Cardwell et al. (2022) [26]
Probability of biphasic reaction following EAI use^b^	0.046	0.037/0.055	Beta (α = 184, β = 3806)	Lee et al. (2015) [27]
Probability of ED visit following severe allergic reaction	Watchful waiting scenario: 0.14 Immediate ED transfer scenario: 1		Fixed	Assumption
Probability of hospitalization following ED visit	0.00168	0.013/0.020	Fixed	Assumption
Annual probability of food allergy fatality	6.9 × 10^− 7^		Beta (α = 100, β = 1.4 × 10^8^)	Greenhawt et al. (2023) [5] Turner et al. (2020) [28]
Increase in food allergy fatality under watchful waiting strategy	10 times		Fixed	Assumption
Food allergy fatality among hospitalized patients	0.0045	0.0036/0.0054	Beta (α = 46, β = 1.0 × 10^4^)	Turner et al. (2020) [28]
Annual probability of food allergy remission for patients 1–6 years	0.058	0.046/0.70	Beta (α = 47, β = 771)	Peters et al. (2022) [30]
**Cost**				
EAI purchase cost, per injector	$95 per injector	$76/$114	Fixed	BC Pharmacare formulary [31] Cardwell et al. (2022) [26]
Probability of using an EAI prior to ED visit	0.54		Beta (α = 7.6, β = 6.5)	Analysis of CCARE registry data
Epinephrine injection cost in ED	$0.8	$0.80/$95	Fixed	Assumption
Epinephrine cost for immediate ED transfer^d^	$52.1	$0.80/$95	Fixed	Assumption
Ambulance cost	$848	$678 /$1017	Gamma (α = 100, β = 8.5)	BC fee schedule [33]
Average annual ED visits in patients with food allergy^e^	0.3 per year		Gamma (α = 0.11, β = 2.7)	Cardwell et al. (2022) [26]
Medical cost of ED visit^e^	$331	$265 /$397	Gamma (α =100, β = 3.0)	CIHI (34) Analysis of CCARE registry data
Average hourly wage for all employees in Canada	$31.37		Fixed	Statistics Canada [36]
Indirect cost of ED visit^f^	$113		Waiting time in ED: Gamma (α = 0.27, β = 13)	CIHI (34) Statistics Canada [36]
Out-of-pocket cost for ED visit	$95		Gamma (α = 100, β = 0.89)	Cardwell et al. (2022) [26]
Daily medical cost of hospitalization^g^	$1866	$1492 /$2339	Total Cost: Gamma (α = 100, β = 37) Days of stay: Lognormal (0.25, 0.99)	Cardwell et al. (2022) [26]
Indirect cost of hospitalization^f^	$251		Fixed	Statistics Canada [36]
Annual direct medical costs of food allergy^e^	$1388	$1110/ $1666	Gamma (α = 100, β = 14)	Cardwell et al. (2022) [26]
Annual out of pocket costs of food allergy^e^	$2577		Gamma (α = 100, β = 24)	Cardwell et al. (2022) [26]
Annual indirect costs of food allergy^e^	$4421		Gamma (α = 100, β = 42)	Cardwell et al. (2022) [26]
Annual direct medical costs of food allergy remission	$569	$455 /$682	Fixed	Fox et al. (2009) [35]
**Utility**				
Food allergy	0.92	0.74/1.0	Beta (α = 7.1, β = 0.62)	Dufresne et al. (2020) [37]
Disutility of severe allergic reaction	-0.090	-0.11/-0.072	Beta (α = 0.17, β = 1.7)	Carroll and Downs (2009) [38]
Food allergy remission	0.93	0.74/1.0	Beta (α = 8.8, β = 0.66)	Guertin et al. (2018) [39] Mittman et al. (1999) [40]

*Abbreviation*: ED, emergency department; BC, British Columbia; CIHI, Canadian Institute for Health Information; CCARE, Cross-Canada Anaphylaxis Registry; CADTH, Canadian Agency for Drugs and Technologies in Health; DSA, deterministic sensitivity analysis; PSA, probabilistic sensitivity analysis

^a^ Probabilities were taken from Cardwell et al. (2022) [26] for patients < 18 years

^b^ Biphasic reaction was defined as the recurrence of symptoms within 72 h of the initial anaphylactic event

^c^ Calculated by adding the probability of not having an EAI (9.4%) to the probability of having a biphasic reaction following EAI use (4.6%)

^d^ Calculated by adding $0.80 for an epinephrine injection in the ED to $95 for using a single EAI before going to the ED multiplied by the probability of using an EAI prior to the ED visit

^e^ Annual ED visit costs were removed from annual direct medical costs, out-of-pocket expenses, and indirect costs by multiplying the average number of ED visits per year by the medical cost per ED visit

^f^ Calculated by multiplying the average waiting time in the ED (3.6 h in the base case) or daily working hours for hospitalization (8 h, fixed parameter) by the average hourly wage ($31.37)

^g^ Daily cost in the base case analysis was calculated by dividing the total hospitalization costs reported in Cardwell et al. [26] by the average length of stay (2.1 days). In probabilistic sensitivity analysis we created separate distributions for total hospitalization costs and days of stay. We divided the samples from each distribution to get daily medical costs of hospitalization

The increased risk of mortality from watchful waiting after at-home EAI use compared to immediate ED transfer following severe allergic reactions is unknown. Therefore, we assumed a tenfold increase in the risk of overall food allergy fatality under watchful waiting (6.9 × 10^− 6^), an assumption that has been used previously [18], and evaluated 100-, 500-, and 1,000-fold mortality increases in scenario analyses. We used 2022 Canadian life tables to determine age-specific all-cause mortality [29].

Patients with food allergies had a 0.058 annual probability of allergy remission between the ages of 1 and 6 years, based on the 29% cumulative probability of resolved peanut allergy observed by Peters et al. [30]. Remission of food allergy did not occur among patients older than 6 years. All probabilities were converted to a daily timescale.

### Resource use and costs

In the base case analysis, we considered only the direct medical costs paid by the healthcare payer. When epinephrine was used in a pre-hospital setting under the watchful waiting strategy, patients were required to purchase a replacement EAI for $95. The EAI purchase cost was determined from patient self-reported data and aligns with the British Columbia Pharmacare formulary [26, 31]. If patients were subsequently transferred to the ED, there was a cost of $0.80 for in-hospital epinephrine injection ($0.74 for the first dose, per *personal communication;* 7.7% of patients with severe reactions require a second dose [32]). In the immediate ED transfer strategy, the total cost for epinephrine was $52.1, determined by adding the cost of epinephrine injection in the ED ($0.80) to an average EAI cost of $51.3 per patient, assuming 54% of patients used a $95 single EAI before going to the ED (unpublished CCARE registry data). Dispensing fees and potential market markups were not included in the medication cost.

The direct medical cost of an ED visit for severe allergic reaction consisted of the land ambulance transfer cost of $848, determined from the BC fee schedule for uninsured individuals [33], and the average medical cost of an ED visit for any cause reported by the Canadian Institute for Health Information [34]. The medical cost of hospitalization for food allergy was $1,866, based on the average daily rate for inpatient allergy admissions reported by Cardwell et al. [26]. The baseline medical costs for patients with food allergy outside of severe allergic reaction were $1,388 per year. This parameter was calculated from the annual costs of food allergy reported by Cardwell et al. [26], with the costs of ED visits removed by assuming an average of 0.3 ED visits per year at a cost of $331 per visit [34], as ED costs were counted separately in our model. The annual medical costs of patients in food allergy remission were $569, which is 59% lower than those with food allergy [35].

In scenario analysis, we included the indirect costs of ED visits and hospitalization from lost productivity, calculated by multiplying the length of stay (3.6 h for an ED visit and 8 h for hospitalization) with a $31.37 average hourly wage reported by Statistics Canada [34, 36]. Out-of-pocket costs for a single ED visit were $95, as reported by Cardwell et al. [26]. Out-of-pocket costs and indirect costs of food allergy outside of severe reaction were also taken from Cardwell et al. [26], with the costs of ED visits removed using the same method as direct medical costs. All costs were adjusted to 2022 CAD using the healthcare component of the consumer price index [36].

### Health utility

The health state utility for patients with food allergy was 0.92, determined using the EQ-5D-5 L generic health-related quality of life questionnaire among Canadian patients aged 0–17 years [37]. A severe allergic reaction incurred a disutility of 0.09 from baseline [38]. Patients with food allergy remission were assigned a utility of 0.93, which is the average health state utility of the Canadian general population aged 12 to 19 years elicited using the Health Utilities Index (HUI) (shown in Table 1) [39, 40].

### Analysis

We calculated the incremental cost per QALY lost through watchful waiting versus immediate ED transfer in a cohort of 10,000 patients with a WTP threshold of $50,000/QALY. In instances where the watchful waiting strategy was cost-saving but resulted in fewer QALYs, ICERs above the WTP threshold were considered cost-effective.

We used one-way deterministic sensitivity analysis (DSA) to assess the impact of a ±20% change in the base case values of the following parameters: the probability of severe allergic reaction, food allergy remission, hospitalization, biphasic reaction and food allergy fatality in hospitalized patients; the cost of an epinephrine, ambulance transport, ED visit, hospitalization, annual direct medical costs, utility for patients with food allergy, food allergy remission and disutility of severe allergic reaction. Epinephrine costs in the ED were varied from their lowest possible cost of $0.80, assuming no patients used an EAI at home and received injections in the ED, to their highest possible cost of $95, assuming all patients used an EAI at home.

We used Probabilistic Sensitivity Analysis (PSA) with a Monte Carlo simulation of 1,000 iterations to evaluate parameter uncertainty. Parameter distributions (shown in Table 1) were derived using the mean and standard deviation reported in the original studies or by assuming a variance-to-mean ratio of 10% when these were not reported. The results of the PSA are presented in a cost-effectiveness plane, where each point represents one iteration of the simulation. The cost-effectiveness acceptability curve shows the proportion of simulation iterations that were cost-effective across a range of WTP thresholds.

We performed the following scenario analyses to assess the robustness of our results through 1,000 PSA iterations: 1) 0% discount rate per annum; 2) 3% discount rate per annum; 3) societal perspective; and increases in food allergy fatality from watchful waiting of 4) 100-fold, 5) 500-fold, 6) 1,000-fold; and 7) decreasing the starting age of patients to zero years.

## Results

The results from the base-case analysis are presented in Table 2. The total discounted costs of the watchful waiting strategy over a 20-year time horizon were $20,641 per patient, compared to $21,798 for the immediate ED transfer strategy. The total discounted QALYs were 15.9026 per patient for watchful waiting and 15.9034 per patient for immediate ED transfer. The incremental cost savings from watchful waiting were $1,157 per patient with a QALY loss of 7.28 × 10^− 4^, resulting in an ICER of $1,589,854 per QALY saved through immediate ED transfer and an INMB of $1,120. The per-patient risk of food allergy fatality over 20-years was 9.2 × 10^− 5^ lower in the immediate ED transfer strategy than in the watchful waiting strategy, which translated to a difference of < 1 death per 10,000 patients over the model time horizon. The incremental cost per death prevented with immediate ED transfer was $12,586,613.

**Table 2:** Base case results

Strategy	Cost ($)	QALYs	Incremental Costs ($)	Incremental QALYs	ICER ($/QALY)	INMB ($)
Watchful waiting	20,641	15.9026	-1,157	-7.28 × 10^− 4^	1,589,854	1,120
Immediate ED transfer	21,798	15.9034	Reference	Reference	Reference	Reference

*Abbreviations*: ICER, incremental cost-effectiveness ratio; INMB, incremental net monetary benefit; QALY, quality-adjusted life year; ED, emergency department

Results are presented per-patient over a 20-year horizon

### Sensitivity analyses

**Fig. 2:**
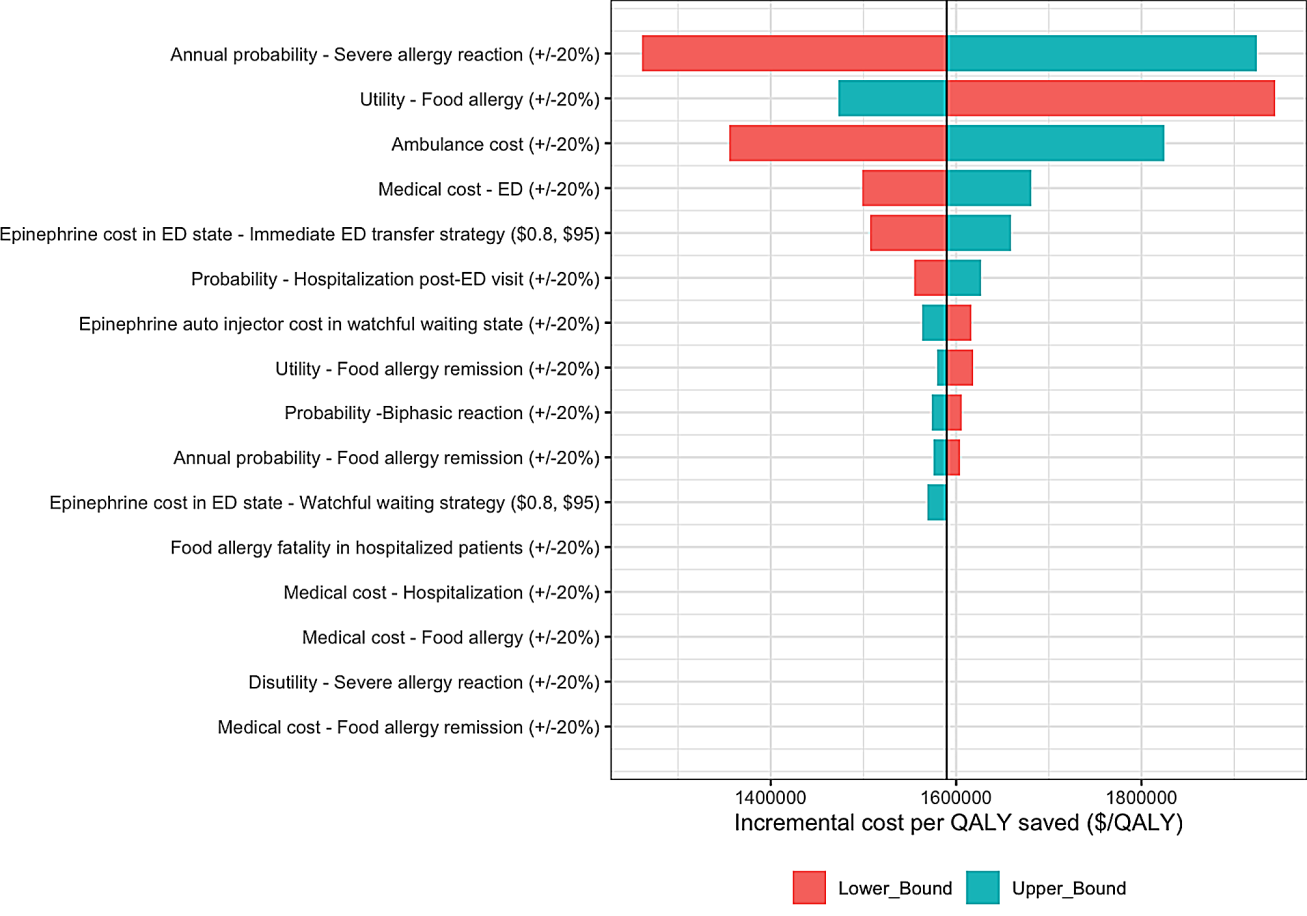
One-way deterministic sensitivity analysis of watchful waiting versus immediate ED transfer. The colored bars indicate the lower (red) and upper (blue) bounds of the parameters in the sensitivity analysis. ED, emergency department; QALY, quality-adjusted life year; ICER, incremental cost-effectiveness ratio

Watchful waiting remained cost-effective compared to immediate ED transfer in one-way sensitivity analysis (Fig. 2). The most influential parameter was the probability of severe allergic reaction; at an annual probability of 7%, the ICER for watchful waiting decreased to $1,261,317 per QALY, indicating a lower value of watchful waiting as the probability of severe allergic reactions decreased. In contrast, when the utility of food allergy outside of severe allergic reactions decreased to 0.74, the value of watchful waiting increased to $1,944,308 per QALY as the utility benefit of higher survival through immediate ED transfer was lower. The most influential cost parameters were the costs of ambulance transfer and ED visits; reducing these costs decreased the value of watchful waiting. The probability of hospitalization, cost of hospitalization, and inpatient risk of mortality had minimal impact on the results owing to the infrequency of hospitalizations.

**Fig. 3:**
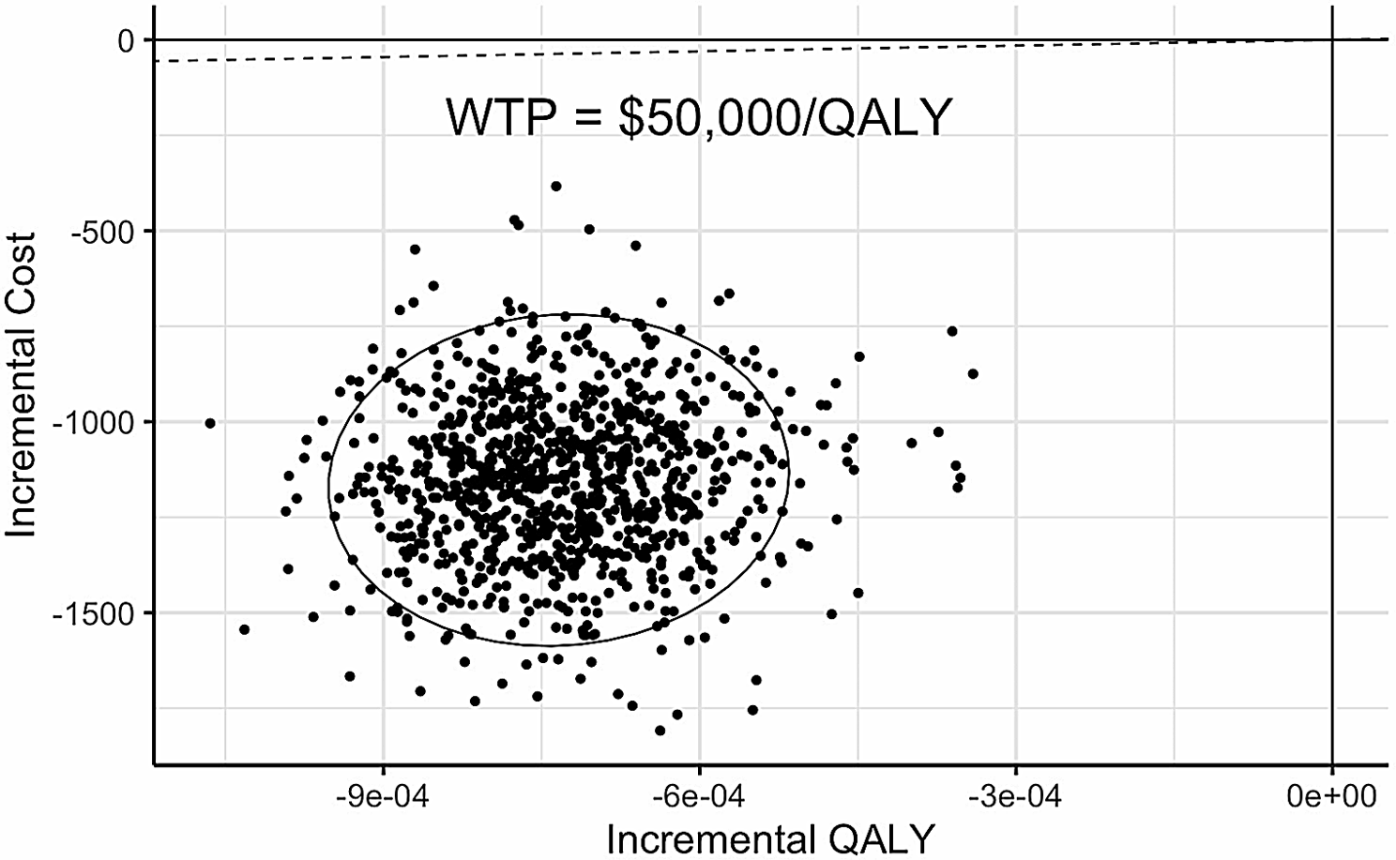
Cost-effectiveness plane from probabilistic sensitivity analysis of watchful waiting versus immediate ED transfer. Each point represents one iteration from the PSA. Points to the right of the WTP threshold (dashed line) are considered cost-effective. Ellipses encompass 95% of iterations

The results of the PSA are shown in Fig. 3. In all PSA iterations, watchful waiting was cost-effective compared to immediate ED transfer at a WTP threshold of $50,000/QALY. The probability that watchful waiting was cost-effective was 100% at WTP thresholds ranging from $0 per QALY to $150,000 per QALY (Supplemental Fig. S1).

Watchful waiting remained cost-effective in all scenario analyses other than with extreme increases in the risk of fatality under watchful waiting of 500-fold and 1,000-fold compared to immediate ED transfer (Table 3). The ICER of watchful waiting increased to $1,887,807/QALY when the indirect and out-of-pocket costs of healthcare use were incorporated in the societal perspective, indicating a higher value of watchful waiting compared to the base case from the healthcare system perspective.

**Table 3:** Scenario analyses

Scenario	Total Cost ($)		Total QALYs		Incremental			
	Watchful waiting	Immediate ED transfer	Watchful waiting	Immediate ED transfer	Costs ($)	QALYs	ICER ($/QALY)	INMB ($)
100-fold increase in food allergy fatality under watchful waiting	20,787	21,949	15.8834	15.8915	-1,163	-8.0 × 10^− 4^	145,104	762
500-fold increase in food allergy fatality under watchful waiting	20,615	21,834	15.8517	15.8920	-1,219	-4.04 × 10^− 2^	30,192	-800
1,000-fold increase in food allergy fatality under watchful waiting	20,363	21,632	15.7981	15.8788	-1,270	-8.07 × 10^− 2^	15,732	-2,765
Societal perspective	111,374	112,752	15.9045	15.9052	-1,378	-7.30 × 10^− 4^	1,887,807	1,341
0% discount rate per annum	3,330	4,655	18.4467	18.4476	-1,324	-8.83 × 10^− 4^	1,499,064	1,280
3% discount rate per annum	2,399	3,411	13.8077	13.8083	-1,013	-6.02 × 10^− 4^	1,682,213	983
Starting age 0 years	20, 733	21, 893	15.9160	15.9167	-1, 160	-7.27 × 10^− 4^	1, 595, 002	1, 087

*Abbreviations*: ED, emergency department ICER, incremental cost-effectiveness ratio; INMB, incremental net monetary benefit; QALY, quality-adjusted life year

## Discussion

Prompt administration of an EAI for food anaphylactic reactions decreases symptoms and the risk of fatality, which can reduce the need for observation in a medical setting. Until recently, management recommendations were to visit an ED for observation regardless of pre-hospital EAI use [14, 15]. We evaluated the cost-effectiveness of watchful waiting for symptom re-occurrence after EAI use in a non-medical setting in patients with rapidly resolved or resolving signs and symptoms, compared to immediate ED transfer regardless of symptom response following EAI use. We found that a strategy of watchful waiting was cost-effective from the healthcare payer perspective, with an incremental cost per QALY saved through immediate ED transfer of nearly $1.6 million. This cost increased to $1.9 million per QALY saved when the indirect costs of lost productivity and out-of-pocket costs from additional ED visits were included. Given the extremely low risk of death from food allergies, substantial medical cost savings could be realized through delayed ED transfer, with minimal risk of adverse events. Further, it is possible that removing the requirement for ED transfer will improve at home EAI use, early resolution of allergic reaction, and improve quality of life as patients feel more empowered to use their EAI at home and less hostage to their allergies.

Our results align with the conclusions of a previous cost-effectiveness analysis of immediate emergency services activation versus watchful waiting following anaphylactic reactions in the US healthcare setting among patients with peanut allergy [18]. Our analysis expanded the study population to include all patients with food allergies, and incorporated real-world Canadian data on the probability of EAI possession and pre-hospital EAI use under the current management strategy of immediate ED transfer. Because patients who did not have access to an EAI transferred to the ED immediately regardless of the management strategy, our results are likely to reflect real-world adherence to management recommendations. Our study was also conducted within the Canadian healthcare context using both direct and indirect food allergy costs. While traditional guidance for ED transfer among patients at high risk of recurrent or non-resolving symptoms remains important, both studies highlight the benefits of promoting initial outpatient treatment with epinephrine. Evidence of the cost-effectiveness of watchful waiting in the U.S. healthcare setting provided important supporting evidence for recently updated U.S. guidelines [13]. Our study contributes similar evidence for the Canadian healthcare system.

Our findings support the findings of other studies endorsing watchful waiting for symptom re-occurrence following EAI use. Fatality in anaphylaxis is an exceptionally rare outcome (overall prevalence of 0.47–0.69 per million persons), and severe biphasic anaphylaxis is even more infrequent (0.5 to 1 death per million person-years) [1, 41–43]. Prompt epinephrine is the main protective factor for these complications [44–46]. Furthermore, the high safety profile of intramuscular epinephrine injection is well-established and adjunct therapies provided in the ED such as antihistamines and steroids have not been shown to reduce the risk of a biphasic reaction nor of fatality [10]. Given these considerations, along with the significant resource use in EDs and, during the COVID-19 pandemic, the increased risk of infection transmission to ED visitors [47–49], the Canadian Society of Allergy and Clinical Immunology has recently recommended watchful waiting following EAI use under a stringent set of circumstances in a shared decision-making approach between patient and clinician [19]. Our analysis supports this recommendation as a cost-saving approach with minimal impact on the quality-adjusted life expectancy of patients with food allergy.

A key assumption in our analysis was that watchful waiting was associated with a tenfold increase in the risk of food allergy fatality compared to immediate ED transfer. Given that data to inform this parameter does not exist, we selected a value at the extreme end of a plausible range. While in hospital monitoring for biphasic reaction following EAI administration is likely to reduce the risk of uncontrolled reaction, the risk of symptom re-occurrence is low and can be appropriately managed with use of a second EAI. In our analysis, watchful waiting remained cost-effective at a 100-fold increase in fatality risk. Only when the fatality risk was increased to implausibly high values of 500-fold and 1,000-fold was watchful waiting less preferred to immediate ED transfer. On the contrary, watchful waiting could decrease the risk of food allergy fatality if pre-hospital use of EAI increases. Recent studies suggest that the requirement to visit an ED following EAI use is a disincentive to early use [12, 50]. In this case, watchful waiting could result in health improvements as well as lower costs. However, any watchful waiting strategy must be accompanied by increased access to EAIs and patient education on the importance of early and proper use.

Our study has several strengths. We used data from CCARE, including several dedicated analyses, to inform the risk of severe allergic reaction, possession of an EAI, and pre-hospital EAI use. CCARE includes all cases of anaphylaxis presenting to the ED of eight major urban hospitals across Canada, and captures pre-hospital management of anaphylaxis at the time of presentation to the ED [24]. We also incorporated evidence on the direct, indirect and out-of-pocket costs of food allergy in the Canadian pediatric population, which were determined from a recent economic burden of disease study among patients recruited from the CCARE registry. Despite the lack of empirical evidence, we robustly analyzed the impact of different fatality risks on the cost-effectiveness of watchful waiting, and provide strong evidence on the value of watchful waiting under plausible assumptions for fatality risk.

Our study has several limitations. First, we did not directly account for an increased risk of hospitalization due to delayed ED transfer after watchful waiting. However, it is important to note that patients in both strategies were assumed to have received epinephrine immediately after anaphylaxis was recognized, but in different locales (ED vs. at-home monitoring for symptom progression). To account for the potential increased risk of food allergy fatality following hospitalization, we assumed ten times increased fatality rate in the watchful waiting group, which we increased to 1,000 times in sensitivity analysis. While not directly accounting for an increased risk of hospitalization under watchful waiting may have underestimated hospitalization costs, the impact on model results is likely to be minimal. Hospitalization costs constituted a small fraction of total costs in the immediate ED transfer strategy and one-way sensitivity analysis confirmed that hospitalization costs had minimal impact on the value of watchful waiting. Second, we did not incorporate the impact of comorbidities (e.g., asthma), history of anaphylactic or biphasic reactions, the patient’s ability to use an EAI correctly and the possibility that patients will be unable to administer a second EAI if the first one fails in our analysis. These factors are likely to affect the risk of food allergy fatality, and anaphylaxis management strategies should be developed through shared decision-making with patients while taking these factors into account. Third, our study did not account for the potential costs of providing EAIs to patients or educating them on proper usage and monitoring following allergic reaction, which are necessary components of a successful watchful waiting strategy. We also did not consider the costs of travel time to the ED in the societal scenario, or incorporate the possibility that patients in the watchful waiting strategy could transfer to the ED when symptoms resolve after EAI injection, which is likely to be a very small proportion of patients.

## Conclusion

Our study suggests that watchful waiting of resolved or resolving allergic reactions is cost saving and has minimal impact on the quality-adjusted life expectancy of patients with food allergy. Watchful waiting for symptom re-occurrence following EAI use, in patients who experience prompt resolution or near-resolution of signs and symptoms, is a cost-effective alternative to universal immediate ED transfer regardless of response to pre-hospital EAI use.

## Electronic supplementary material

Below is the link to the electronic supplementary material.

Supplementary Material 1

## Data Availability

The data used and generated during the current study are included in the article, and codes are publicly available at https://github.com/resplab/Allergy_EAI_CEA.
