# Supplementary material for: Cost-effectiveness of watchful waiting versus immediate emergency department transfer after epinephrine autoinjector use in Canada
Source: Allergy Asthma Clin Immunol. 2025 Jan 22;21:5. doi: 10.1186/s13223-025-00951-w (PMC11755952; doi:10.1186/s13223-025-00951-w)
Supplement: Supplementary file 1 — Supplementary Material 1 [file 13223_2025_951_MOESM1_ESM.docx]

**Table S1.** Consolidated health economic reporting standards (CHEERS) checklist

| **Topic** | **No.** | **Item** | **Location where item is reported** |
| --- | --- | --- | --- |
| **Title** | 1 | Identify the study as an economic evaluation and specify the interventions being compared. | Page 1 |
| **Abstract** | 2 | Provide a structured summary that highlights context, key methods, results, and alternative analyses. | Page 3 |
| **Introduction** | | | |
| **Background and objectives** | 3 | Give the context for the study, the study question, and its practical relevance for decision-making in policy or practice. | Page 4-5 |
| **Methods** | | | |
| **Health economic analysis plan** | 4 | Indicate whether a health economic analysis plan was developed and where available. | NA |
| **Study population** | 5 | Describe characteristics of the study population (such as age range, demographics, socioeconomic, or clinical characteristics). | Page 6 |
| **Setting and location** | 6 | Provide relevant contextual information that may influence findings. | Page 6 |
| **Comparators** | 7 | Describe the interventions or strategies being compared and why chosen. | Page 6 |
| **Perspective** | 8 | State the perspective(s) adopted by the study and why chosen. | Page 6 |
| **Time horizon** | 9 | State the time horizon for the study and why appropriate. | Page 6 |
| **Discount rate** | 10 | Report the discount rate(s) and reason chosen. | Page 6 |
| **Selection of outcomes** | 11 | Describe what outcomes were used as the measure(s) of benefit(s) and harm(s). | Page 6 |
| **Measurement of outcomes** | 12 | Describe how outcomes used to capture benefit(s) and harm(s) were measured. | Page 6 |
| **Valuation of outcomes** | 13 | Describe the population and methods used to measure and value outcomes. | NA |
| **Measurement and valuation of resources and costs** | 14 | Describe how costs were valued. | Page 6 |
| **Currency, price date, and conversion** | 15 | Report the dates of the estimated resource quantities and unit costs, plus the currency and year of conversion. | Page 6 |
| **Rationale and description of model** | 16 | If modeling is used, describe in detail and why used. Report if the model is publicly available and in which it can be accessed. | Page 6 |
| **Analytics and assumptions** | 17 | Describe any methods for analyzing or statistically transforming data, any extrapolation methods, and approaches for validating any model used. | Page 6 -10 |
| **Characterizing heterogeneity** | 18 | Describe any methods used for estimating how the results of the study vary for subgroups. | NA |
| **Characterizing distributional effects** | 19 | Describe how impacts are distributed across different individuals or adjustments made to reflect priority populations. | NA |
| **Characterizing uncertainty** | 20 | Describe methods to characterize any sources of uncertainty in the analysis. | Page 11-12 |
| **Approach to engagement with patients and others affected by the study** | 21 | Describe any approaches to engage patients or service recipients, the general public, communities, or stakeholders (such as clinicians or payers) in the design of the study. | NA |
| **Results** | | | |
| **Study parameters** | 22 | Report all analytical inputs (such as values, ranges, references) including uncertainty or distributional assumptions. | Page 7-11 |
| **Summary of main results** | 23 | Report the mean values for the main categories of costs and outcomes of interest and summarize them in the most appropriate overall measure. | Page 12 |
| **Effect of uncertainty** | 24 | Describe how uncertainty about analytical judgments, inputs, or projections affect findings. Report the effect of choice of discount rate and time horizon, if applicable. | Page 13-14 |
| **Effect of engagement with patients and others affected by the study** | 25 | Report on any difference patient/service recipient, general public, community, or stakeholder involvement made to the approach or findings of the study. | NA |
| **Discussion** | | | |
| **Study findings, limitations, generalisability, and current knowledge** | 26 | Report key findings, limitations, ethical or equity considerations not captured, and how these could affect patients, policy, or practice. | Page 14-17 |
| **Other relevant information** | | | |
| **Source of funding** | 27 | Describe how the study was funded and any role of the funder in the identification, design, conduct, and reporting of the analysis. | Page 1 |
| **Conflicts of interest** | 28 | Report authors conflicts of interest according to journal or International Committee of Medical Journal Editors requirements. | Pag 1 |

From: Husereau D, Drummond M, Augustovski F, et al.^1^ Consolidated Health Economic Evaluation Reporting Standards 2022 (CHEERS 2022) Explanation and Elaboration: A Report of the ISPOR CHEERS II Good Practices Task Force. Value Health 2022;25. doi:[10.1016/j.jval.2021.10.008](https://doi.org/10.1016/j.jval.2021.10.008).

**Table S2.** Calculation of the probability of hospitalization and fatality outside of hospital used in the model

| **Parameter name** | **Probability** | **Daily probability adjustment^a^** | **Parameter abbreviation** | **Reference** |
| --- | --- | --- | --- | --- |
| Annual food allergy fatality with immediate ED transfer strategy | 6.9×10^-7^ | 1.89×10^-9^ | Fatality_ED_ | Greenhawt et al. (2023)  Turner et al. (2020) |
| Annual food allergy fatality with watchful waiting strategy | 6.9×10^-6^ | 1.89×10^-8^ | Fatality_ww_ | Assumption |
| Annual probability of severe allergic reaction following accidental exposure | 0.087 | 2.49×10^-4^ | P_severe_ | Analysis of CCARE registry data |
| Food allergy fatality among hospitalized patients | 0.0045 | Not applicable | Fatality_hosp_ | Turner et al. (2020) |
| Probability of ED transfer with watchful waiting strategy | 0.14 | Not applicable | P_ED_ | Assumption |

^a^ Annual probabilities were converted to daily probabilities using the formula $P_{daily}=1-{(1-P_{annual})}^{\frac{1}{365}}$. Daily probabilities were used in the following calculations.

The probability of hospitalization (P_hosp_) for patients in the ED transfer state is given by:

$$P_{hosp}= \frac{{Fatality}_{ED}}{P_{severe} \times{Fatality}_{hosp}}=0.00168$$

Food allergy fatality outside of hospital in the watchful waiting state (Fatality_ww state_) is given by:

$$\mathrm{Fatality}_{ww state}=\left( \frac{{Fatality}_{ww}}{P_{severe}}-P_{ED}\times P_{hosp}\times{Fatality}_{hosp} \right)\div\left( 1-P_{ED} \right)=$$

$8.69\times{10}^{-5}\left( daily \right)$ = $0.031$ (annually)


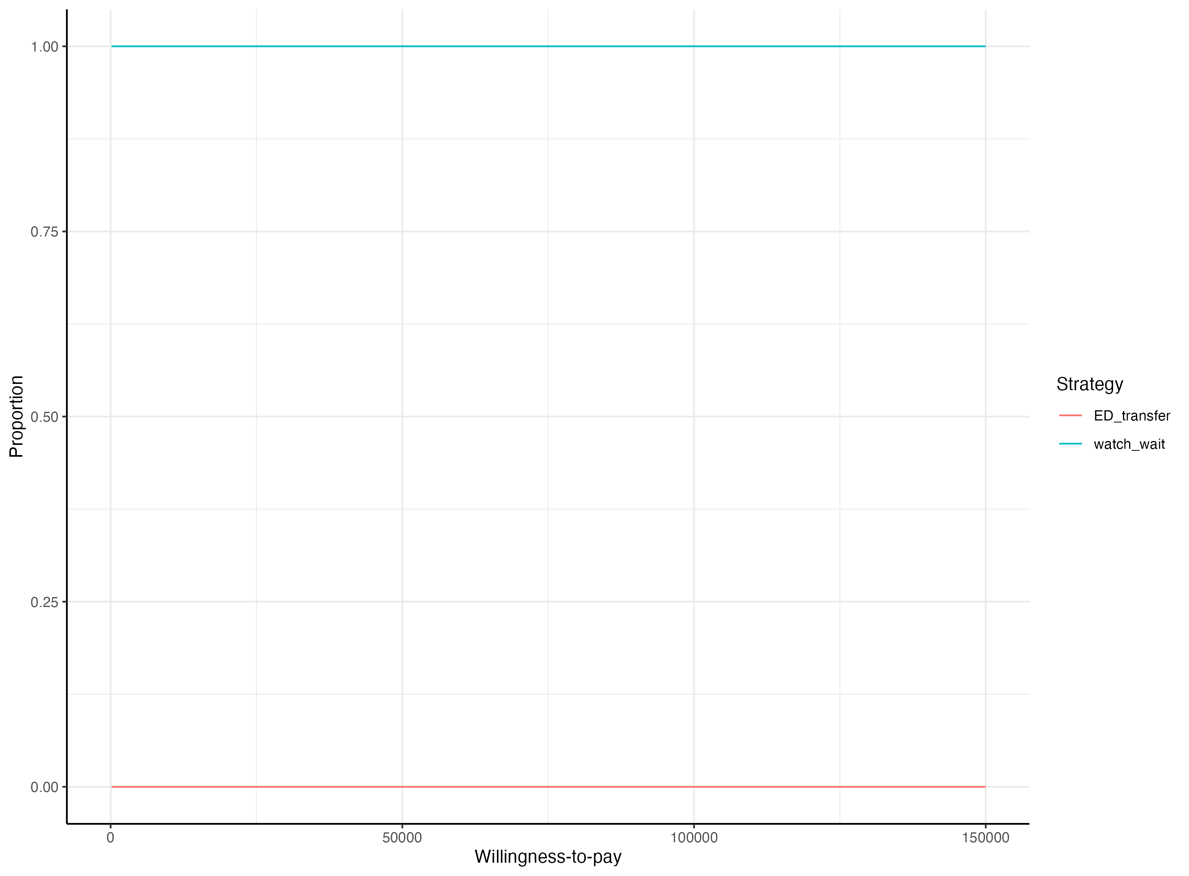


**Figure S1.** Cost-effectiveness acceptability curve showing the proportion of scenarios in which watchful waiting (blue line) was cost-effective compared to immediate ED transfer (red line) at various willingness to pay thresholds.
